# Retraction: Construct comprehensive indicators through a signal extraction approach for predicting housing price crises

**DOI:** 10.1371/journal.pone.0282628

**Published:** 2023-03-01

**Authors:** The PLOS ONE Editors

Following the publication of this article [1], concerns were raised regarding peer review integrity.

Upon review of the submission history for the manuscript, the editors found strong indications that the peer review process was compromised.

These concerns were not resolved following discussion with the authors.

In light of these concerns, the *PLOS ONE* Editors retract this article. We regret that the issues in this article were not identified prior to publication.

TL did not agree with the retraction and stands by the article’s findings. YX, YM, ZZ and JL either did not respond directly or could not be reached.
